# See Me Smoke-Free: Protocol for a Research Study to Develop and Test the Feasibility of an mHealth App for Women to Address Smoking, Diet, and Physical Activity

**DOI:** 10.2196/resprot.5126

**Published:** 2016-01-21

**Authors:** Peter Giacobbi Jr, Melanie Hingle, Thienne Johnson, James K Cunningham, Julie Armin, Judith S Gordon

**Affiliations:** ^1^ Sport Sciences, Epidemiology West Virginia University Morgantown, WV United States; ^2^ Nutritional Sciences University of Arizona Tucson, AZ United States; ^3^ Computing Sciences University of Arizona Tucson, AZ United States; ^4^ Family and Community Medicine University of Arizona Tucson, AZ United States

**Keywords:** smoking cessation, mhealth, diet, exercise, imagery, cell phone, handheld app

## Abstract

**Background:**

This paper presents the protocol for an ongoing research study to develop and test the feasibility of a multi-behavioral mHealth app. Approximately 27 million women smoke in the US, and more than 180,000 women die of illnesses linked to smoking annually. Women report greater difficulties quitting smoking. Concerns about weight gain, negative body image, and low self-efficacy may be key factors affecting smoking cessation among women. Recent studies suggest that a multi-behavioral approach, including diet and physical activity, may be more effective at helping women quit. Guided imagery has been successfully used to address body image concerns and self-efficacy in our 3 target behaviors—exercise, diet and smoking cessation. However, it has not been used simultaneously for smoking, diet, and exercise behavior in a single intervention. While imagery is an effective therapeutic tool for behavior change, the mode of delivery has generally been in person, which limits reach. mHealth apps delivered via smart phones offer a unique channel through which to distribute imagery-based interventions.

**Objective:**

The objective of our study is to evaluate the feasibility of an mHealth app for women designed to simultaneously address smoking, diet, and physical activity behaviors. The objectives are supported by three specific aims: (1) develop guided imagery content, user interface, and resources to reduce weight concern, and increase body image and self-efficacy for behavior change among women smokers, (2) program a prototype of the app that contains all the necessary elements of text, graphics, multimedia and interactive features, and (3) evaluate the feasibility, acceptability, and preliminary efficacy of the app with women smokers.

**Methods:**

We created the program content and designed the prototype application for use on the Android platform in collaboration with 9 participants in multiple focus groups and in-depth interviews. We programmed and tested the application’s usability with 6 participants in preparation for an open, pre- and posttest trial. Currently, we are testing the feasibility and acceptability of the application, evaluating the relationship of program use to tobacco cessation, dietary behaviors, and physical activity, and assessing consumer satisfaction with approximately 70 women smokers with Android-based smart phones.

**Results:**

The study was started January 1, 2014. The app was launched and feasibility testing began in April 1, 2015. Participants were enrolled from April 1-June 30, 2015. During that time, the app was downloaded over 350 times using no paid advertising. Participants were required to use the app “most days” for 30 days or they would be dropped from the study. We enrolled 151 participants. Of those, 78 were dropped or withdrew from the study, leaving 73 participants. We have completed the 30-day assessment, with a 92% response rate. The 90-day assessment is ongoing. During the final phase of the study, we will be conducting data analyses and disseminating study findings via presentations and publications. Feasibility will be demonstrated by successful participant retention and a high level of app use. We will examine individual metrics (eg, duration of use, number of screens viewed, change in usage patterns over time) and engagement with interactive activities (eg, activity tracking).

**Conclusions:**

We will aggregate these data into composite exposure scores that combine number of visits and overall duration to calculate correlations between outcome and measures of program exposure and engagement. Finally, we will compare app use between participants and non-participants using Google Analytics.

## Introduction

This paper presents the protocol for an ongoing research study to develop and test the feasibility of a multi-behavioral mobile health app (mHealth app), See Me Smoke-Free, aimed at helping women smokers to quit. Each year, about 180,000 women in the U.S. die from smoking-related illnesses [1]. In 1987, lung cancer surpassed breast cancer as the leading cause of cancer death among U.S. women [2]. Lung cancer causes as many deaths as breast and gynecological cancers combined [2]. Smoking causes about 80% of Chronic Obstructive Pulmonary Disease (COPD) deaths among women annually, and women smokers are more than 10 times more likely to die from COPD than nonsmokers [3]. Women who smoke double their risk of coronary heart disease, and are at increased risk for developing cancer and fractures [1]. Almost 46% of women smokers try to quit annually [4]. The Lung Health Study found that women may benefit from quitting more than men [5].

### Special Issues Related to Quitting Among Women

A large body of empirical evidence suggests that women are less successful at quitting smoking [6] and are more likely to relapse than men [7]. This may be because of concerns about gaining weight, lack of social support, stress and depressed mood [8], and physiological symptoms related to the menstrual cycle [6,7,9-11]. Biology may also be a factor as women report greater withdrawal symptoms when they quit smoking than men [12-14], and withdrawal symptoms may be affected by menstruation [11,15,16]. In addition, research indicates that nicotine replacement therapy may be less effective for women [9,17-19].

Women gain 8-10 pounds on average after quitting smoking [20-22]. About 25% of female quitters gain <5 pounds, 50% gain 5-15 pounds, and 25% gain >15 pounds [23-25]. Thus, it is no surprise that half of women smokers report concerns about weight gain as a barrier to quitting [6,26], and most women quitters who relapse identify weight gain as a factor [7]. About half of women smokers report weight control as a reason for smoking [27], and 52% identify weight gain as the reason for relapse [28]. Weight concerns cross age and racial boundaries, but younger, Caucasian women report the most weight concern [29,30]. Two meta-analyses indicated that pre-smoking cessation concerns about weight gain and pre-cessation BMI (Body Mass Index) did not appear to reduce the amount of weight gained when quitting [31,32].

Women who are highly concerned about their weight are less likely to contemplate quitting, and are more likely to smoke as a means to control weight [26]. Several studies suggest that women smokers endorse a thinner body image and are less satisfied with their bodies than never-smokers [33,34]. Weight-concerned women report overall body image as a factor in deciding whether to quit, and in relapse after quitting [26]. A recent meta-analysis suggests that incorporating a personalized weight management program may reduce weight gain without undermining cessation, and recommended further research in this area [32]. Incorporating a body image component into a cessation intervention for women is warranted. Studies have shown that these smokers may be more successful in quitting if they can attain a more realistic body image [34-36].

### Addressing Multiple Risk Factors

A small but growing body of literature suggests that a simultaneous, rather than sequential, approach may be more successful for decreasing weight and sedentary activity, and may contribute to successful smoking cessation among women [32,37-39]. According to a recent meta-analysis by Spring and colleagues [38] that included 10 randomized controlled trials, combined smoking plus weight treatment produces significantly higher short-term abstinence and significantly lower weight gain than did smoking treatment alone. The studies included in this meta-analysis showed continued advantage to the multi-behavioral approach on abstinence after 6 months, although the effect on weight control was no longer significant [38]. Other smoking cessation interventions have shown that participation in structured exercise can be an effective smoking cessation strategy compared to general wellness (control) groups although mixed results have been observed [40-42]. While these studies did not target body image per se, meta-analytic results have shown that exercise does improve body image irrespective of changes in fitness or body weight [43]. Thus, the inclusion of exercise behavior as part of a cognitive intervention that targets body image concerns may be an effective way to increase smoking abstinence in women who smoke [44-48].

### Theoretical Framework and the Use of Guided Imagery

#### Body Image

Cognitive behavioral theory (CBT) predicts that cognitive biases lead to overvaluation of body size and shape, internalization of thin body ideals, body image dissatisfaction, and maladaptive weight control practices among women (eg, smoking, dieting) [49]. Compared to nonsmokers, women who smoke are less satisfied with their bodies, more concerned about appearance, and have lower self-esteem [50]. The rate of smoking among female college students is related to body image [32,37-39]. These findings suggest that targeting body image concerns may be an effective way to address smoking as part of a multi-behavior intervention. One such study found that a CBT approach, including mindfulness exercises, to address body image produced significantly greater abstinence than a weight management intervention [35].

#### Concerns About Weight Gain

Studies show that weight concerns play an important role in the smoking behavior of women [34,51]. Compared to nonsmokers, women who smoke are more concerned about becoming overweight [50]. Smoking among women is also related to weight concerns, dieting behavior, and disordered eating symptoms [27,35,52-55]. Concerns about weight also persist after quitting [28]. Weight gain after quitting results from increased dietary caloric intake and decreased metabolic rate [56,57], therefore most behavioral interventions attempt to decrease food intake and/or increase physical activity [6,20,40,42,58,59]. According to a recent meta-analysis of tobacco cessation interventions that aim to prevent weight gain, one of the most effective interventions to increase abstinence and reduce weight gain focused on a cognitive intervention to minimize concern about weight [6].

#### Self-Efficacy

Self-efficacy refers to the belief or confidence in one’s ability to successfully execute a given behavior and is frequently considered one of the central determinants involved in the behavior change process [60,61]. According to self-efficacy theory, individuals' beliefs about their capacity to change a behavior causally influence the outcome when they engage in a behavior change attempt [62]. Self-efficacy theory has been studied widely in the smoking cessation literature. For decades this theory has been used to help identify how confidence in one’s ability to quit smoking influences smoking cessation behavior. When people feel confident in their ability to quit smoking, they are more likely to plan to quit [63,64]. This literature suggests that self-efficacy is positively correlated with making plans to quit, especially in the short-term.

### Guided Imagery as an Intervention Strategy

**Figure 1:**
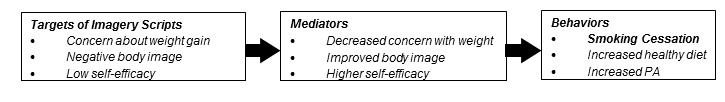
Project targets, mediators and goal behaviors.

Guided imagery is a form of mind-body therapy that involves controlled visualization of specific mental images, and overlaps with mindfulness meditation [65]. Both guided imagery and mindfulness have the core feature of focusing awareness on attention [66]. As shown in Figure 1, guided imagery scripts will target concerns about weight gain, perceptions of body image, and self-efficacy to quit smoking, eat a healthy diet, and engage in physical activity.

It is generally accepted that body image disturbances are associated with disordered eating etiology. Studies have shown that mental imagery is an effective way to address eating disorders, concerns about weight gain, and muscle dysmorphia [67-69]. Meta-analytic reviews have consistently documented the effectiveness of CBT in the treatment of eating disorders [70,71]. Given that mental imagery is an important part of CBT therapy, and this therapeutic modality can effectively address negative body image, we predicted that guided imagery scripts would change women smokers' concerns about body weight and image. Further empirical justification for this prediction is readily gleaned from published studies focused on diet, exercise, and smoking cessation among other health behaviors.

Guided imagery has been shown to improve self-efficacy across a broad range of behaviors, including performance of novel motor tasks [72], prevention of falls with older adults [73], postsurgical functional outcomes [74], and pain management in persons with fibromyalgia [75]. More specific justification for targeting the theorized mediators of smoking cessation using guided imagery is provided in Figure 1.

Separate lines of inquiry support the use of mental imagery to enhance physical activity (PA) behavior, improve diet, and smoking cessation [40,58,76-81]. Several recent studies suggest that guided imagery and other mindfulness-based interventions can be effective in assisting smokers to quit [82]. In this study, participants who used an audio CD-based guided imagery intervention were significantly more likely to be abstinent or to have reduced their level of smoking at all follow-up points than control subjects [82]. Another study using imagery resulted in reduced cravings with smokers [80]. Another recent study found that a mindfulness-based intervention attenuated relationships between negative affect and smoking urges, as well as between negative affect and body dissatisfaction [83].

Cross-sectional and intervention studies have clearly documented the impact of guided imagery on exercise behavior [84]. A recent RCT demonstrated that guided imagery scripts effectively increased exercise self-efficacy with sedentary women [78]. The first author (PRG) has conducted numerous studies showing that mental imagery is associated with exercise behavior [85-88]. These studies have shown that individuals who use mental imagery focused on fitness or health goals and exercise technique report greater amounts of exercise than those who do not use imagery. Another randomized trial that used guided imagery among other intervention components resulted in increased exercise behavior after 18-months follow-up as compared to control group participants [89]. In another study, experimental group participants showed increased self-determined exercise behavior after a 10-week imagery intervention as compared to controls [90].

A growing body of literature suggests that guided imagery can be effective at improving dietary behaviors [77,91,92]. One recent study used mental imagery to target implementation intentions in order to increase fruit consumption [91]. Participants randomized to a mental imagery condition targeting implementation intentions demonstrated significantly higher self-reported fruit consumption after 7-days follow-up compared to control, implementation intentions, and goal intentions conditions [91]. These results suggest that mental imagery targeted to specific behavioral intentions may be an effective way to increase goal attainment with dietary behavior. Other studies have also shown that mental imagery is associated with reduced craving or actual consumption of food [77,92,93].

The dual function framework of mental imagery guided the development of the imagery scripts [94]. This framework has recently been supported with psychometric analyses [86]. The dual function framework predicts that mental imagery serves cognitive (eg, skill development) and motivational functions. The guided mental imagery scripts involved images of successfully executing a behavior (eg, walking for exercise) and enhanced self-efficacy, regulation of urges or emotions (eg, anxiety) linked to body image, and goal achievement (eg, losing or maintaining weight).

### Potential Impact of Mobile Health Apps

A recent study by Spring and colleagues [39] provided evidence to support the use of mobile technology to successfully address multiple behavior changes in diet and physical activity. There is enormous potential reach of mobile apps. As of February 2012, 88% of U.S. adults used a mobile phone, and 53% of these are mobile phones with enabled Internet capacity [95]. In 2012, the top 3 selling mobile platforms in the U.S. were Android (20% of mobile phone owners), iPhone (19%), and Research in Motion (6%) [95]. There are >500,000 mobile software apps (apps) available for the iPhone, Android, and Blackberry devices, which collectively have been downloaded >25 billion times. In 2012, approximately 19% of mobile phone owners had at least one health app on their phone and 52% used their devices to search for health information, and this figure is expected to grow exponentially [95].

Very little research has tapped the great potential of interactive technologies to address multiple risk factors [95]. A mobile phone-based approach offers reach as well as convenience. To our knowledge, the proposed study is the first of its kind to specifically target these underserved smokers using a mobile health platform. If successful, a mobile health app using guided imagery would have broad applicability beyond this intervention and population. The lessons learned in the proposed project could be applied to developing mobile health apps for other chronic conditions with multiple risk factors and the populations at risk for developing those conditions.

The systematic development and evaluation of a mobile health app is novel. According to Abroms and colleagues [96], the currently available mobile apps for tobacco cessation are not evidence based and do not provide effective treatment. Backinger and Augustson [97] called for the systematic development of mobile apps in this area to provide efficacious treatment for tobacco cessation. Our proposed study would be the first of its kind to create and test a mobile app to address smoking, diet and physical activity in an adult population.

Successful studies of multiple risk factor interventions have traditionally been conducted in in-person, individual, or group settings [97]. A growing mismatch between the demand for health-related services and supply has burdened our health care system [98-103]. The use of mobile technologies can help to reduce some of this burden by replacing a proportion of face-to-face encounters with a model that emphasizes patient self-management, independent of time and place. Mobile technologies allow users 24/7 access to information and resources, as well as simple tools for self-monitoring, an important component of any health behavior change intervention. Mobile apps are also highly customizable, and can be easily configured by the individual patient to provide tailored feedback in support of behavior change. These features increase the likelihood that critical information and feedback is delivered to the patient when they need it most. Further, the flexibility of mobile apps allow capture of individual patterns of behavior (eg, cravings); data which can then be correlated with diet and physical activity behaviors (and other data) to better understand the factors related to smoking.

## Methods

The proposed project produced an interactive mobile phone app to deliver a guided imagery intervention targeting concerns about body weight, body image, and self-efficacy in order to increase tobacco cessation, healthy eating, and physical activity. We completed the following: Aim 1) created program content and designed the functionality of the prototype app for use on the Android platform, in collaboration with 9 participants in multiple focus groups (several participants attended more than 1 focus group) and 1 in-depth interview; Aim 2) programmed and tested the app’s usability with 6 participants; and Aim 3) we are currently pilot testing the feasibility and acceptability of the app, evaluating the relationship of program use to tobacco cessation, dietary behaviors, and physical activity, and assessing consumer satisfaction with 73 weight-concerned women smokers who own an Android-based mobile phone. In a future R01, we will test the efficacy of the app in a randomized controlled trial.

### Project Time Line

This project occurred in several stages over 2 years. Development of the content and user interface occurred over the first 6 months of Year 1. Based on the results of focus group testing, the prototype mobile app was programmed, user testing and program refinement then occurred over the last half of Year 1. The feasibility trial is currently taking place (Year 2), and includes programming of the informed consent and study data collection tools, participant recruitment, data collection, data analysis, report writing, and development of the Manual of Procedures for the future randomized trial.

### Program Development Process

During Stage 1, the team collected content materials, reviewed other health-related cell-phone apps, and outlined the desired interface design, functionality, and content for the prototype app. Then, the team drafted content elements for the intervention (see Textbox 1). Five guided imagery audio files were created (1 introductory file, 3 behavior-specific files, and 1 general file) and 3 resource pages related to smoking cessation, diet, and physical activity were developed. These resources pages addressed the common barriers experienced by women smokers when quitting (eg, cessation resources, weight-loss resources, physical activity resources). The app provided suggestions for addressing these barriers and a direct link to each resource, based on user input. We also provided a daily diary/tracking calendar to track use of the guided imagery scripts, tobacco use/cessation variables (eg, cravings, withdrawal symptoms and number of cigarettes smoked), diet variables (eg, increased fruit/vegetable intake), and physical activity variables (eg, frequency, intensity and type of exercise). These user inputs were designed to tailor messages around the benefits of continued practice with the scripts and provide suggestions for alternative strategies (eg, behavioral support for cessation, including a “one touch” call to their local tobacco quitline). The technology was developed to promote user input and interactivity, and time spent listening to mental imagery files. Many program elements, such as the tracking calendar and data collection and feedback, were refined based on responses from focus group participants.

**App components.**

*Guided imagery audio files*

1. Introduction to Guided Imagery

2. Be Smoke-Free

3. Eat Well

4. Get Moving

5. Feel Fantastic

*Resources for tobacco cessation*

1. Direct connect to tobacco quitlines

2. Links to evidence-based information

*Daily diary*

1. Smoking

2. Eating 5 fruits and vegetables

3. Getting 30 minutes of exercise

*Tailored feedback based on user input*

1. Awards for meeting goals

Motivational messages

In Stage 2, the programming team prepared functional specifications for the software platform and designed the interactive features. These features included the User Profile function that facilitates participants’ tailoring of the program (eg, set a quit date, set time for delivery of guided imagery files). In addition, the entire team designed the overall infrastructure to accommodate user registration, baseline surveys, send/receive functions and within-app navigation. All design features were incorporated into a graphic storyboard that described the prototype and illustrated the app’s important functions, then presented to focus group participants for their review. Participants helped refine each of the proposed program components or suggest additions. Participants provided feedback on length of imagery audio files, narrator voice, resources and information, elements of the user interface, content and timing of messages. In Stage 3, based on feedback from focus groups, we revised some of the program components while in stage 4, program assets elements (eg, text, graphics, images, resource materials) were finalized and programmed into the app.

**Figure 2:**
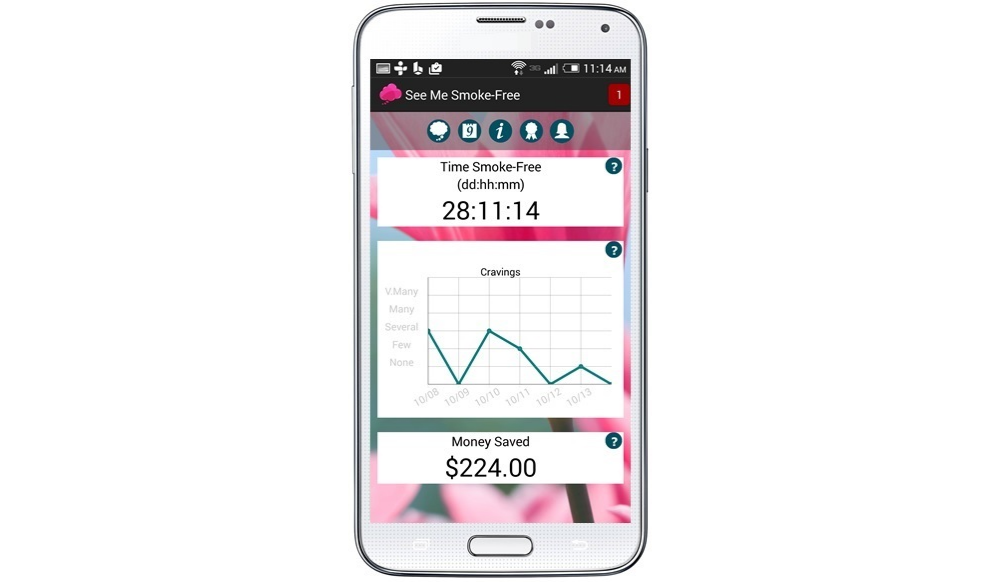
See Me Smoke Free app home screen.

Stage 5 focused on development of the “Alpha” version of the app. Programmers created rapid prototypes of the app that allowed the project team to review and confirm that app functionality, content and design were consistent with the overall goals of the program. After internal testing of the Alpha version, we recruited groups of participants for usability testing, which yielded a final set of changes prior to feasibility testing. The app was fully automated and programmed using the Android application programming interface (API). The app takes full advantage of all of the functionality made available by the Android API including push notifications, saving and retrieval of local data, sending/retrieval of data over the network, and interfacing with the project database. The tight integration of data and programming allowed us to tailor app content (eg, deliver content based on quit date or program use) as well as unobtrusively track all usage details and user choices. The developers built the app to accommodate expansion of the content and the migration of the app to other mobile platforms in future projects (Figure 2).

### Evaluation Overview

We used qualitative and quantitative methods and conducted focus group and usability testing with 9 participants in 4 focus groups and 7 in-depth interviews (several participants attended more than 1 focus group or interview), as well as feasibility and consumer satisfaction testing of the prototype product with 73 participants.

### Participant Recruitment and Eligibility

Participants were female cigarette smokers who were interested in quitting. Additional eligibility requirements include having smoked for ≥ 1 year, being at least 18 years old, having Internet and e-mail access, and speaking English.

Local participants were recruited via University social media and other websites, Craigslist, recruitment flyers in health care clinics and other community locations, and news coverage in local media outlets. Potential participants for focus and user groups were contacted via phone by trained project staff who described the research study and invited individuals to participate. Participants recruited for focus groups and user groups resided in the greater Tucson, Arizona area. Focus group participants were considered eligible if they owned any type of mobile phone.

Participants recruited for the feasibility test could be located anywhere in the United States. Local and national press coverage about the study and no-cost Facebook, Twitter, and other social media sites helped spread the word about recruitment for the study. Informed consent and all data collection were conducted via the mobile app. After a participant was consented, project staff collected contact information, including the participant’s cell phone number, email, and mailing address. Usability test and feasibility test participants needed to own an Android phone to be eligible to participate. All participants received a US $50 check for completing the study.

### Formative and Process Evaluation

#### Focus Groups

A total of 9 women smokers (7 White, 1 Hispanic/Latino, and 1 White/Native American) participated in four 1.5-hour focus groups and 1 in-depth interview to offer input about the prototype design. During the focus groups, participants were asked to discuss topics that directly informed the refinement of our app with respect to program presentation, features (eg, daily diary), script content, and potential tailoring of content based on differences in body image [104-106]. We followed recommended guidelines for focus groups [107,108]. Session transcripts were analyzed for salient constructs, issues, and language use to inform further program development. The first and second focus groups reviewed the 5 imagery scripts developed by the team. After the first 2 focus groups, the imagery scripts were refined and the prototype design modified for presentation to the third and fourth groups for input.

#### Usability Testing

A total of 6 participants were recruited to test user interactions with the Alpha prototype. Testing usability with 5 users per population of interest is generally deemed sufficient based on established guidelines [109]. As participants used the Alpha prototype, they were asked to think aloud, verbalize their reactions as they viewed screens, and describe their navigational choices as they made them. Participants performed each of the major tasks the app supports. Interviewers took detailed notes so that comments could be matched to screens or technical functions that elicited feedback. Participant responses were characterized as positive or negative reactions to the features and functions, and difficulty or facility with using the app or interface. Usability testers also completed the 10-item adaptation of Brooke’s widely-used and validated System Usability Scale and a consumer satisfaction questionnaire used in the principal investigator’s (JG) previous research [109]. After each tester provided feedback, revisions were made to the Alpha prototype, and additional user tests were conducted. Usability testing took approximately 1½ hours per session.

#### Feasibility and Satisfaction Testing

The feasibility and acceptability of the final Alpha prototype is being evaluated in an open, pre/post trial with 73 women smokers with Android mobile phones.

### Study Procedures

Participants downloaded the app from the Google Play store. After launching the app, participants were asked if they would like to participate in research. If so, they were screened for eligibility, and eligible respondents provided informed consent and completed baseline assessments. Project staff were available to assist if a participant experienced difficulty, and any technical problems encountered by participants were documented. Participants were asked to use the app “most days” for 30-days. During this period, participant use and engagement data were collected. Prompts for the follow-up assessments were delivered and completed via the mobile phone or via a weblink.

### Data Collection

#### Demographic and Descriptive Variables

Demographic information collected at baseline included year of birth, ethnicity, and socio-economic status (SES).

#### Tobacco Use History, Current Use Patterns, and Nicotine Dependence

Use of all tobacco products were assessed using a series of questions that have been standardized and employed in previous studies [110-112], and the Fagerström Tolerance Nicotine Dependence Scale [113].

#### Body Mass Index

Self-reported height and weight will be converted to body mass index (kg/m^2^).

#### Concern about Weight Gain

We used items from the Weight Control Smoking Scale [114] and the Concern about Post-Cessation Weight Gain Scale [115] to measure concerns about weight, use of smoking to control weight, and concerns about gaining weight as a result of quitting smoking. Both measures have been used extensively and have demonstrated reliability and validity [114].

#### Self-Efficacy

We assessed the degree of confidence to prevent weight gain after quitting smoking with the *Weight Efficacy after Quitting Scale*, and self-efficacy for quitting with the 15-item version of the Condiotte & Lichtenstein Confidence Questionnaire [116].

#### Body Image

The Multidimensional Body-Self Relations Questionnaire [117] provides a standardized, attitudinal assessment of body image. We chose the 7-item Appearance Evaluation Subscale [117,118] which measures feelings of physical attractiveness and satisfaction or dissatisfaction with one’s appearance [119,120].

#### Diet

To assess the daily number of fruit servings and vegetable servings, the 2-item interactive version of the National Cancer Institute Fruit and Vegetable Scan was used [121]. Also used were several questions from the Dietary Screener Questionnaire used in the National Health and Nutrition Examination Survey (NHANES).

#### Physical Activity

We assessed physical activity with the Godin Leisure-Time Exercise Questionnaire (LTEQ) [122]. The LTEQ has a long history of use [123] and is simple to administer using a mobile app. It is also sensitive to mild, moderate, and strenuous exercise and is scored in two ways: total number of minutes of activity or a metabolic equivalents (METS) expenditure estimate.

#### Tobacco Use Outcome Measures

As recommended by the Society for Research on Nicotine and Tobacco [124] and Velicer and Prochaska [125] we used several self-report measures, including prolonged abstinence and point prevalence.

#### Withdrawal and Cravings Symptoms

All participants were asked to rate their experience with withdrawal symptoms and cravings using a 5-point rating scale of severity 3 times per day at random intervals [126]. To do so more often would have created a confounding effect on the intervention, and may also have increased response burden to unacceptable levels.

#### App Satisfaction and Acceptability

At follow-up, we will use Tullis and Stetson’s 8-item adaptation [127] of Brooke’s widely used System Usability Scale to rate the usability of our app [109]. We also asked participants about their satisfaction with the program, perceived usefulness of information, relevance to smoking cessation efforts, whether they would recommend the program to others.

#### App Use

We monitored (1) the length of time participants interact with the app, (2) the number of screens they visit within the app, (3) use of the daily diary, and (4) the number of links they click on while using the app.

### Data Analysis

#### Focus Group and Usability Testing

Interviews conducted during focus groups were audio-recorded, and cross-referenced with notes taken by trained staff. User testing interviews were conducted by 2 trained staff, with one in charge of interacting with the participant and the other responsible for detailed note-taking. Transcripts and notes were analyzed by several members of the project team with extensive expertise in analyzing focus group and user group data for common themes, changes to the program content and/or functionality, and suggestions for improvement.

#### Feasibility and Acceptability Testing

The feasibility and acceptability of the program is being evaluated in an open trial with 73 participants. A pre/post design will evaluate participants’ reported usability and satisfaction with the product, reported abstinence from smoking, and actual use of the program. While this design does not control for potential threats to internal validity, it will allow for the initial evaluation of the program’s impact on cessation. Threats to internal validity will be addressed in a subsequent RCT.

The feasibility of the program will be demonstrated by the achievement of the following benchmarks: (a) recruiting at least 50 women to use the app regularly within a 3-month window, (b) a high level of app usage by the participants (eg, 80% of participants using the program regularly during the data collection phase), and (c) based on our previous experiences of program usage, a retention rate of at least 70% at the 3-month assessment. Acceptability will be defined as a high degree of consumer satisfaction (eg, mean usability and satisfaction ratings >4 on a 5-point scale).

#### Tobacco Use, Diet, and Physical Activity

We will assess the absolute cessation rate of participants, the smoking rate for nonquitters and the number of quit attempts. Paired *t*-tests will be used to evaluate change in each of these cigarette consumption measures from baseline to 30 days post, and again from baseline to 90 days post. With 50 participants, significance level = 0.05, there is 0.80 power to detect change of *d*=0.66, a medium effect size. We will also use multilevel modeling for repeated measures to examine change in each food/beverage consumption measure across all 3 waves (baseline, 30 days and 90 days), with level-1 units consisting of the repeated measures for each participant, and the level-2 unit being the participant. (Note that multilevel modeling is tolerant of missing waves of data.) For each wave, we will also construct composite measures of concern about weight gain (a composite of 3 Likert-type items), perceived self-efficacy regarding smoking cessation (5 Likert-type items), body image (7 Likert-type items), mental imagery (4 Likert-type items), and diet (4 items assessing juice, lettuce, vegetable and water consumption). Body weight/BMI and the frequency and intensity of exercise will be measured as well. Multilevel modeling will be used to examine changes in these measures across the 3 waves. Finally, multilevel modeling will also be used to examine whether weight gain, perceived self-efficacy regarding smoking cessation, body image, diet, body weight/BMI and exercise have an association with the absolute cessation rate of participants, the smoking rate for nonquitters and the number of quit attempts.

#### Program Use and Engagement

The See Me Smoke-Free (SMSF) app allows monitoring of all app usage by study participants through the intervention database. However, we will also use Google Analytics which allows us to track a limited number of app functions for both participants and nonparticipants. Participants will have all app usage tracked in the intervention database with regular synchronizations from the SMSF app. The analytics provided by Google include anonymized information for the users, differentiating by a textual tag if the user is a participant or nonparticipant. It tracks the following actions: visit the app home screen; set or update a quit date; start or finish listening to an audio file. Thus such statistics are available daily for study participants and nonparticipants. We will examine individual metrics (ie, visit duration, number of type of screens viewed, change in usage patterns over time), and interactive activities (eg, answer daily questions) engaged in for study participants. We will also aggregate these data into composite exposure scores that combine number of visits and overall visit duration to calculate correlations between outcome and measures of program exposure and engagement [128,129]. Finally, we will compare app use on visits to the home screen, setting or updating a quit date, and listening to the audio files between participants and nonparticipants using the data from Google analytics.

## Results

The study was started January 1, 2014. The app was launched and feasibility testing began in April 1, 2015. Participants were enrolled from April 1-June 30, 2015. During that time, the app was downloaded over 350 times using no paid advertising. Participants were required to use the app “most days” for 30 days or they would be dropped from the study. We enrolled 151 participants. Of those, 78 were dropped or withdrew from the study, leaving 73 participants. We have completed the 30-day assessment, with a 92% response rate. The 90-day assessment is ongoing. During the final phase of the study, we will be conducting data analyses and disseminating study findings via presentations and publications.

## Discussion

### Preliminary Results

This paper presents the protocol used in an ongoing study to develop and test a guided imagery intervention targeting smoking, diet and physical activity among women. The multi-behavioral intervention is delivered via an mHealth app, called See Me Smoke-Free. We used an iterative approach to development, including focus groups, individual interviews, and user testing, to develop the app content, functionality, and user interface. The app was successfully deployed to the Google Play store, and downloaded hundreds of times. We learned many lessons about the challenges faced when developing technology to be delivered using the Android platform, recruiting participants with no in-person contact, and gathering data from multiple sources.

#### Future Directions

We are currently preparing 2 manuscripts that describe in detail our recruitment and retention strategies and our development process. We are in the final stages of data collection and have begun data analyses. The results of these analyses will be described in a future outcome paper.

## Abbreviations

API: application programming interface
BMI: body mass index
CBT: cognitive behavioral theory
COPD: chronic obstructive pulmonary disease
LTEQ: Godin Leisure-Time Exercise Questionnaire
METS: metabolic equivalents
NHANES: National Health and Nutrition Examination Survey
PA: physical activity
RCT: randomized controlled trial
SMSF: See Me Smoke-Free
